# Primary Hepatic Lymphoma Mimicking a Hepatocellular Carcinoma in a Cirrhotic Patient: Case Report and Systematic Review of the Literature

**DOI:** 10.1155/2018/9183717

**Published:** 2018-04-10

**Authors:** Ali Bohlok, Thierry De Grez, Fikri Bouazza, Roland De Wind, Melody El-Khoury, Deborah Repullo, Vincent Donckier

**Affiliations:** ^1^Service de Chirurgie, Institut Jules Bordet, Université Libre de Bruxelles (ULB), Bruxelles, Belgium; ^2^Service de Gastroentérologie, CHR Sambre et Meuse, Namur, Belgium; ^3^Service d'Anatomie Pathologique, Institut Jules Bordet, Université Libre de Bruxelles (ULB), Bruxelles, Belgium

## Abstract

**Introduction:**

Primary hepatic lymphomas (PHLs) are rare liver tumors, frequently misdiagnosed preoperatively. As these tumors could be successfully treated with chemotherapy, their early recognition is essential, potentially, to avoid useless surgery. We report on the case of a cirrhotic patient with hemochromatosis who presented a PHL, initially diagnosed as a hepatocellular carcinoma (HCC), and we analyze recent data from the literature on this subject.

**Case Presentation and Review of the Literature:**

A 45 mm liver tumor was found is a 68-year-old man with alcohol cirrhosis and hemochromatosis. At imaging, the diagnosis of HCC was suspected according to vascular characteristics and the presence of cirrhosis. FDG PET scan showed a solitary hypermetabolic liver tumor. Tumor markers were negative. Surgery consisted in left lateral hepatectomy. At pathology, the diagnosis of the primary hepatic marginal zone B cell lymphoma of mucosa-associated lymphoid tissue (MALT) type was demonstrated. Twenty-two articles reporting 33 cases of true PHL of MALT type were found. Presentation lacked specific symptoms (70% asymptomatic). Half of patients were suspected to have other etiologies of liver mass (HCC, intrahepatic cholangiocarcinoma), and thus diagnosis was established postoperatively. In the patient, diagnosis was made by preoperative biopsy, and chemotherapy was first-line treatment.

**Discussion:**

Preoperative diagnosis of PHL, and particularly of primary hepatic MALT lymphoma, is challenging. This case illustrates that PHL remains to be considered among the differential diagnosis of isolated solid liver tumors. Further, it indicates that biopsy could be still indicated in case of suspected HCC in cirrhotic patients, particularly in the presence of unusual findings such as the combination of a FDG PET scan positive tumor in the absence of elevated alpha-fetoprotein.

## 1. Introduction

Primary hepatic lymphoma (PHL) represents less than 10% of space occupying focal hepatic lesion [1]. Among lymphoma, PHLs represent 0.4% of all extranodal lymphomas and 0.016% of all non-Hodgkin lymphomas [2]. Primary hepatic marginal zone B cell lymphoma of mucosa-associated lymphoid tissue (MALT) is extremely rare, representing 3% of PHL [3]. Because of this rarity and in the absence of specific symptoms and imaging characteristics, the early recognition of PHL is a challenge and most of the diagnoses are established on operative specimen [4]. Multiple treatment modalities depending on the timing of diagnosis of PHL include chemotherapy and surgery associated or not with chemotherapy [5]. Therefore, preoperative recognition of PHL could be critical, as it may avoid unnecessary liver resection or reduce the extent of liver resection in case of response to preoperative chemotherapy [6, 7]. Herein, we present a case of surgically resected primary hepatic MALT lymphoma mimicking HCC on preoperative imaging, a review of literature of primary hepatic MALT lymphoma, and further discuss on the need for additional tools to establish such diagnosis preoperatively. In addition, we review recent data from the literature in order to identify potential recommendations for the diagnosis and the treatment in these cases.

## 2. Materials and Methods

We performed a systematic review of the literature using two databases (PubMed and Scopus). In the PubMed search, we used the following search terms (“Liver Neoplasms” [Mesh]) AND “Lymphoma, B-Cell, Marginal Zone” [Mesh]. Free terms included (“Liver OR hepatic” AND “Lymphoma, B-Cell, Marginal Zone”). In the Scopus database search, we used the terms TITLE-ABS-KEY (“Lymphoma, B-Cell, Marginal Zone” AND ” liver” OR” hepatic”).

The search strategy had no publication date or publication type restriction. In addition, the reference lists of relevant reviews or included articles were also searched to find other eligible studies. We included only studies published in English.

Study characteristics such as patient age, sex, and presenting symptoms, associated liver disease and lesions, the presence of cirrhosis, associated liver lesions, timing of diagnosis (preoperative biopsy or postoperatively), type of treatment, and outcome were evaluated.

## 3. Case Presentation

**Figure 1:**
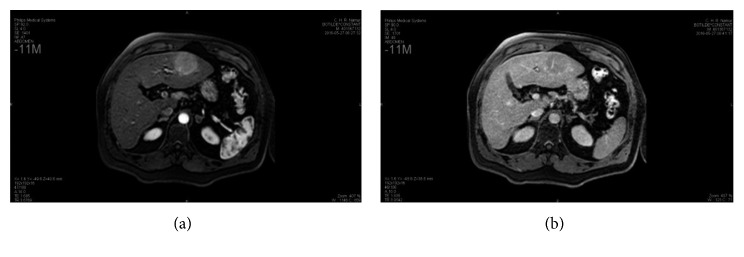
Liver MRI showing the segment III lesion, enhancing at the arterial phase (a) with rapid washout on the portal phase (b).

**Figure 2:**
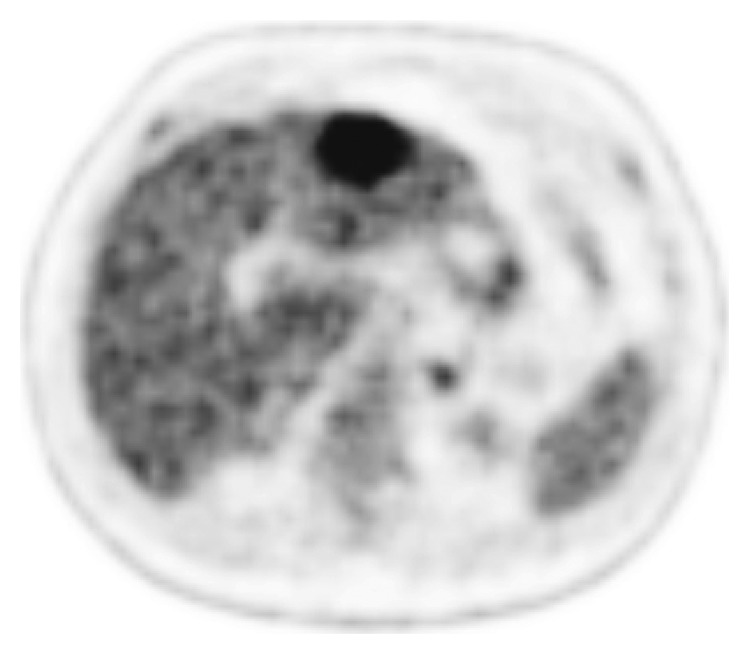
FDG PET-CT showing the hypermetabolic segment III liver lesion (SUV max: 7.3).

**Figure 3:**
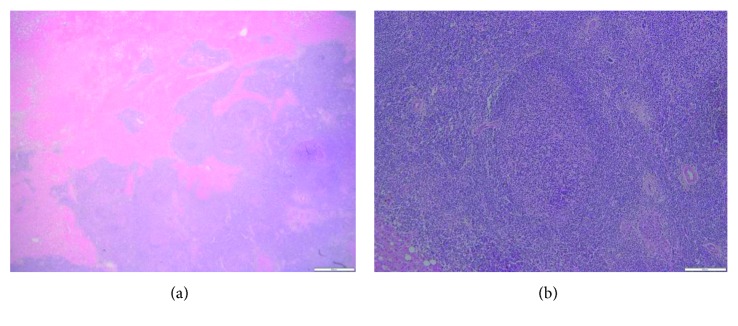
(a) Lymphoid infiltration (bluish discoloration) in hepatocellular parenchyma and (b) germinal center (×100).

**Figure 4:**
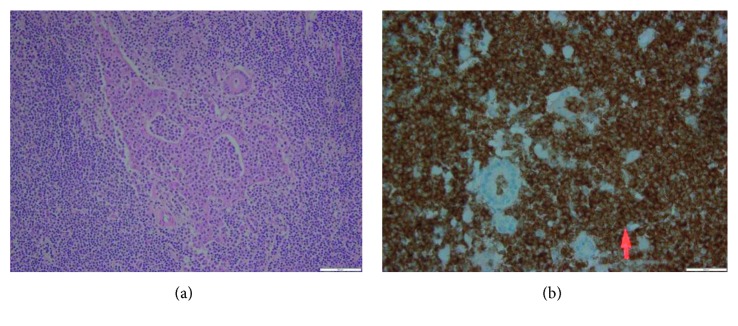
Lymphoepithelial complex in hematoxylin eosin stain (×200) (a) and CD20 staining (×400) (b).

A 68-year-old male patient with hemochromatosis and Child A cirrhosis due to alcohol consumption was referred to our department for a liver tumor. The patient is known to have a Child A cirrhosis without any previous episode of liver decompensation. Physical examination was normal, and laboratory tests showed a minimal elevation of transaminases levels. Serologies for hepatitis B and C and tumor markers, including alpha-fetoprotein (AFP) and CA19.9, were negative. Contrast-enhanced liver MRI showed a 46 mm tumor in segment III with arterial wash in and portal phase washout (Figures 1(a) and 1(b)). Liver morphology, and particularly irregular hepatic surface, confirmed the presence of cirrhosis. On this basis, the diagnosis of hepatocellular carcinoma (HCC) on cirrhotic liver was proposed. A FDG PET scan showed a hypermetabolic liver tumor in segment III, without extrahepatic lesion (Figure 2). Bone scintigraphy and chest CT scan showed no distant metastases, and gastroscopy showed no esophageal varices. Accordingly, a left lateral liver R0 resection was performed. Postoperative course was uneventful, and the patient was discharged at day 4 after surgery. Pathological examination demonstrated the diagnosis of MALT lymphoma (Figures 3(a) and 3(b)). Small-to-middle-sized lymphocytes formed lymphoepithelial lesions on some bile capillaries (Figure 4(a)). Immunohistochemical stains were positive for CD20 (++) (Figure 4(b)), CD3 (+), CD5 (+), CD43 (+), CD10 (+), Ki67 (10%) anti BCL-6 (+), MUM-1 (+), and CD138 (+), whereas they were negative for CD1. PCR amplification showed monoclonal Ig *λ* and IG *κ* (kappa). The pathology of the nontumor liver confirmed the presence of cirrhosis.

The patient's disease was classified as Ann Arbor stage IE (stage I extranodal), and no adjuvant therapy was proposed.

## 4. Systematic Literature Review

### 4.1. Study Selection

**Figure 5:**
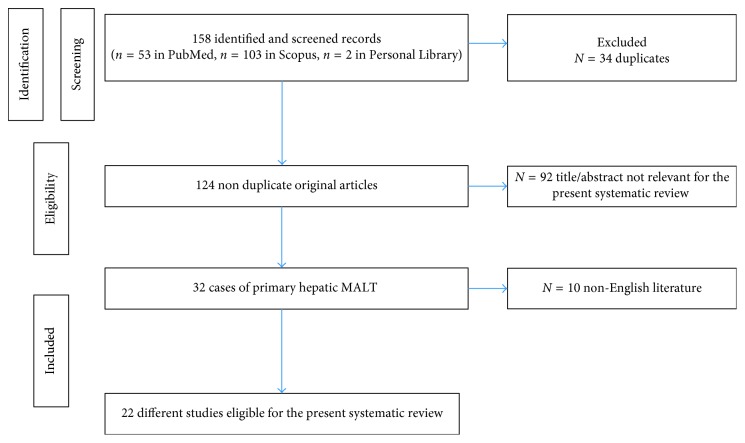
Prisma guidelines for the selection of eligible articles of primary hepatic MALT lymphoma.

A total of 158 articles were identified from the PubMed and Scopus. After removing duplicated articles, 124 articles were assessed further assessment. A total of 92 articles were excluded on the basis of the titles and the abstracts. Only true cases of primary hepatic MALT were included (Ann Arbor stage IE). All cases of extrahepatic biliary tract MALT were excluded. Of the remaining 32 articles, only 22 articles had full text published in English. After full-text review of these remaining articles, they were all eligible and included in the systematic review (Figure 5).

### 4.2. Patient and Tumor Characteristics

A total of 22 articles (Table 1) reporting 33 cases of primary hepatic MALT were found [8–29]. The statistical analysis of all patient characteristics is summarized in Table 1.

There were 17 male (51.5%) and 16 female (48.5%) patients with a mean age of 61.7 years (range 36 to 85 years). Twenty-one patients (63.6%) were asymptomatic at presentation, 3 had abdominal pain (9.1%), and 2 had elevated liver enzymes (6.1%). Fifteen patients had associated liver disease. Cirrhosis was documented in 7 cases (21.2%) including hepatitis C and B and primary biliary origins. Two patients (6.1%) had associated HCC lesion at the time of diagnosis. MRI and CT scan reports were available in only 6 and 7 cases, respectively. PHL of MALT type was described in most cases to be hypointense T1 and iso- or hyperintense T2 on MRI and hypodense with variable enhancement on contrast-enhanced CT. The number of hepatic MALT lesions ranged between 1 and 6 with a mean of 1.34 and median of 1. The mean size of the lesions was 35.61 mm (15–90 mm). Detailed FDG PET report was available in only 1 case series of 5 patients, suggesting that these tumors only show minimal increase in FDG uptake, as indicated by mean SUV of 5.5 (3.5–8.2) [28]. In another case, the tumor was reported as hypermetabolic but no SUV max result was described [26].

Primary hepatic MALT was diagnosed based on biopsy in 15 cases (45.5%) and postoperatively in 16 (48.5%) cases. Treatment options varied depending on the timing of diagnosis. Exclusive chemotherapy (most had R-CHOP), and exclusive radiotherapy were administered in 6 patients (18.2%) and 2 patients (6.1%), respectively. Tumors were resected in 23 patients (70%); of these, 4 were diagnosed posthepatic transplantation (12.1%), and the remaining had surgical resection of the tumor.

Adjuvant therapy was administered in 8 patients and consisted of chemotherapy, antiviral treatment, *Helicobacter pylori* eradication treatment, radiotherapy in 5 (15.2%), 1 (3%), 1 (3%), and 1 (3%), respectively. The follow-up period ranged between 9 months and 5 years with a median of 24 months. At the end of follow-up, only 3 relapses (9.1%) were noted and they were treated by chemotherapy (R-CHOP) in 2 patients [24] and radiotherapy in 1 patient [28]. In operated patients, no correlation could be established between the risk of recurrence and the use of adjuvant chemotherapy or radiotherapy.

## 5. Discussion

In this case, the initial presentation was highly suggestive for the diagnosis of HCC, according to vascular imaging features, the presence of cirrhosis, and for the diagnosis of hemochromatosis. On this basis, in the presence of Child A cirrhosis without significant portal hypertension, surgical resection was decided, without preoperative biopsy [30, 31]. On pathological specimen, the unexpected diagnosis of PHL of MALT subtype was found. In fact, such diagnosis could have change dramatically the treatment plan if it has been obtained preoperatively. Indeed, these tumors are chemosensitive and recent studies reported major response rates using rituximab with cyclophosphamide, doxorubicin, vincristine, and prednisone (R-CHOP), leading to complete remission in more than 80% of the cases [5–7]. The clinical presentation of PHL is variable from incidental discovery in asymptomatic patient, to nonspecific symptoms such as fatigue, night sweats, weight loss, night fever or jaundice, abdominal pain, and hepatomegaly in case of massive liver involvement [32]. The laboratory tests are poorly contributive, and tumor markers, including CEA, AFP, and CA19.9, are usually negative [33]. Among PHL, hepatic MALT lymphoma may arise as solitary or multiple lesions, with variable imaging characteristics, including enhancement on the arterial phase and late washout, as in HCC [34] The definitive diagnosis of MALT is based on pathology, showing CD20 positivity on immunohistochemistry and the presence of the lymphoepithelial complexes [35]. As a consequence, PHLs are rarely diagnosed preoperatively and most frequently confounded with other malignant lesions [7]. In the present case, however, the absence of elevated AFP together with a positive FDG PET scan might have served as an alert, to consider alternative diagnosis to HCC. Separately, neither normal AFP level nor hypermetabolic tumor on FDG PET scan may exclude the diagnosis of HCC. However, while the overall sensitivity of FDG PET scan for the diagnosis of HCC is approximately of 50% [36], it has been shown that HCC could be FDG positive mostly in case of large tumor, superior to 50 mm, corresponding to moderately or poorly differentiated tumor and associated with high AFP levels [37]. Accordingly, in the present case, even if not recommended in current algorithms for tumor superior to 20 mm with characteristic vascular imaging behavior on cirrhotic liver [30, 31] a liver biopsy could have been indicated, leading eventually to avoid first-line liver resection.

In conclusion, primary hepatic MALT lymphoma is an extremely rare tumor with poorly contributive clinical, laboratory, and imaging findings. Early recognition is infrequent, and the definite diagnosis is mostly based on histopathologic examination of operative specimen. The present case illustrates that this diagnosis should be kept in mind and that preoperative biopsy could be still indicated, particularly when the diagnosis of HCC is suspected but in presence of unusual findings such as the combination of positive FDG PET scan with normal AFP level.

According to literature review, the preoperative diagnosis of this tumor remains a challenge, due to the scarcity of specific clinical and imaging signs [8, 11, 14, 24]. In the cases where diagnosis could be established preoperatively, patients should be orientated first to chemotherapy (CHOP) as this treatment could be curative [13, 24, 25]. In the frequent cases where the diagnosis is established postoperatively and when surgical resection has been radical, the role for adjuvant chemotherapy remains unclear [8, 12, 14, 15, 17].

## Figures and Tables

**Table 1:** Patient characteristics.

Characteristics	
Number	
Age	61.67 range(36–85) median: 61 y
Sex	
Male	17 (51.5%)
Female	16 (48.5%)
Presenting symptom	
Asymptomatic	21 (63.6%)
Abdominal pain	3 (9.1%)
Elevated liver enzymes	2 (6.1%)
Generalized weakness	1 (3%)
Cough	1 (3%)
NA	5 (15.2%)
Preexisting liver disease	
HCV	7 (21.2%)
HBV	6 (18.2%)
Primary biliary cirrhosis	1 (3%)
Old HAV	1 (3%)
None	16 (48.5%)
NA	2 (7.1%)
Cirrhosis	
Yes	7 (21.2%)
No	26 (78.8%)
Associated HCC	2 (6.1%)
Number	1.34 range(1–6) median 1
Size (cm)	35.61 mm range(15–90) median: 30 mm
Available MRI description	6 (18.2%)
Available CT scan description	7 (21.2%)
Available FDG PET	5 (15.2%)
Mean SUV max	5.5 (3.5–8.2)
Diagnosis	
Preoperative biopsy	15 (45.5%)
After surgery	16 (48.5%)
NA	2 (6.1%)
Type of treatment	
Resection	12 (36.4%)
Transplantation	4 (12.1%)
Chemotherapy	6 (18.2%)
Radiotherapy	2 (6.1%)
Surgery + chemotherapy	5 (15.2%)
Surgery + antiviral treatment	1 (3%)
Surgery + radiotherapy	1 (3%)
No treatment	2 (6.1%)
Outcome	
Disease free survival at the last follow-up (9 months–5 years)	21 (63.6%)
Relapse at 30–51 months	3 (9.1%)
Death	2 (6.1%)
Lost to follow-up	2 (6.1%)
NA	5 (15.2%)

NA: non available; HCV: hepatitis C virus; HBV: hepatitis B virus; HAV: hepatitis A virus; HCC: hepatocellular carcinoma; MRI: magnetic resonance imaging; CT: computed tomography; FDG PET: 2-deoxy-2[F-18]fluoro-D-glucose positron emission tomography; SUV: standardized uptake value.
